# Laboratory Testing to Inform Antimicrobial Use for Bovine Respiratory Disease: Perceptions of Canadian Feedlot Veterinarians

**DOI:** 10.3390/vetsci12050409

**Published:** 2025-04-27

**Authors:** Olufunto O. Adewusi, Candace I. J. Nykiforuk, Cheryl L. Waldner, Nathan E. N. Erickson, Sheryl P. Gow, Simon J. G. Otto

**Affiliations:** 1 HEAT-AMR (Human-Environment-Animal Transdisciplinary Antimicrobial Resistance) Research Group, School of Public Health, University of Alberta, Edmonton, AB T6G 1C9, Canada; 2 School of Public Health, University of Alberta, Edmonton, AB T6G 1C9, Canada; 3 Centre for Healthy Communities, School of Public Health, University of Alberta, Edmonton, AB T6G 1C9, Canada; 4 Policy, Location, and Access in Community Environments Research Lab (PLACE), School of Public Health, University of Alberta, Edmonton, AB T6G 1C9, Canada; 5 Department of Large Animal Clinical Sciences, Western College of Veterinary Medicine, University of Saskatchewan, Saskatoon, SK S7N 5B4, Canada; 6 Centre for Foodborne, Environmental, and Zoonotic Infectious Diseases, Public Health Agency of Canada, Saskatoon, SK S7N 5B4, Canada

**Keywords:** antimicrobial stewardship, Canadian feedlot veterinarians, prudent antimicrobial use, judicious antimicrobial use, laboratory testing, bovine respiratory disease, antimicrobial resistance

## Abstract

Timely, accurate, and reliable laboratory tools are important for antimicrobial stewardship (AMS) in food animal production. The objectives of this study were to (1) identify factors that influence live animal respiratory sample collection for laboratory testing and (2) describe the potential for integration of laboratory testing of samples from live animals into an AMS strategy for bovine respiratory disease (BRD) management in Canadian feedlots. Utilizing focused ethnography, virtual key informant interviews were conducted with eight feedlot veterinarians from Alberta, Ontario, and Saskatchewan, Canada. Thematic analysis revealed four themes: (1) lived experience of feedlot veterinarians with laboratory testing for BRD pathogens and antimicrobial resistance (AMR), (2) evidence-informed BRD management that integrates multiple data sources and their components, (3) organizational factors that affect the uptake and use of laboratory tests, and (4) the role of laboratory testing to support AMS in BRD management. While sample collection and laboratory testing are commonly used for BRD and AMR research and surveillance, they are not routinely applied in everyday BRD management. Veterinarians identified key challenges with laboratory testing, including turnaround time, the need for clear benefits, practical implementation, and effective communication with feedlot managers. Laboratory testing must provide valuable, actionable insights to encourage its adoption in Canadian feedlot operations. If successful, such testing could support AMS and be used to justify antimicrobial use if this were to become a regulatory or market requirement.

## Simple Summary

This study explores how laboratory testing can support responsible antimicrobial use as part of the management of respiratory diseases in Canadian feedlot cattle. Researchers interviewed feedlot veterinarians from across Canada to understand what influences their decision to collect respiratory samples from live animals and how laboratory results can be integrated into respiratory disease management strategies. The findings highlight that while sample collection and laboratory testing are commonly used for research and monitoring, they are not routinely applied in everyday respiratory disease management. Veterinarians identified key challenges with laboratory testing, including turnaround time, the need for clear benefits, practical implementation, and effective communication with feedlot managers. Laboratory testing must provide valuable, actionable insights to encourage its adoption in Canadian feedlot operations. If successful, such testing could support antimicrobial stewardship and responsible antimicrobial use. This could help to meet future regulations or market demands. This research is valuable because it supports responsible antimicrobial use in food animal production, which can help reduce the risk of antimicrobial resistance and improve animal health and welfare.

## 1. Introduction

Antimicrobial use (AMU) in humans and animals can select for antimicrobial resistance (AMR) in bacteria that pose a threat to human and animal health [1,2,3]. The enormity of the implications of AMR prompted a consensus by the World Health Organization (WHO) to develop a global action plan in 2015 [4] and create an antimicrobial stewardship (AMS) framework in 2017 [5] to be replicated in member countries. Antimicrobial stewardship encompasses multiple approaches to sustain the efficacy of existing antimicrobials and minimize the emergence of resistance through evidence-based AMU [6]. An AMS strategy encompasses actions aimed at the 5 R’s: Responsibility, Reduction, Refinement, Replacement, and Review [7]. One of the specific objectives of the WHO AMS framework was to foster increased investment in developing more effective laboratory tools [4], the results of which can inform AMS efforts under the 5 R’s.

In western Canadian beef feedlots, bovine respiratory disease (BRD) is an important cause of morbidity and mortality and accounts for over 90% of injectable AMU by veterinarians [8]. Bovine respiratory disease is a multifactorial disease that affects the respiratory system of cattle, causing coughing, fever, difficulty breathing, and sometimes death [9]. Several pathogens (bacteria and viruses) have been associated with BRD; however, the disease is often exacerbated by stress-induced immunosuppression that comes with weaning, auction processing, transport to the feedlot, and mixing of calves from different origins [9]. Common bacterial pathogens include *Mannheimia haemolytica*, *Pasteurella multocida*, *Histophilus somni*, *Mycoplasma bovis*, and *Bibersteinia trehalose* [10]. In the BRD complex, the four common viruses that can play an important role in pathogenesis include bovine herpesvirus 1, bovine parainfluenza virus 3, bovine viral diarrhea virus, and bovine respiratory syncytial virus [11,12].

Most Canadian cattle are fed to finish in the provinces of Alberta (60.1%), Saskatchewan (9.9%), and Ontario (19.9%) [13]. Feedlots in Alberta and Saskatchewan range in bunk capacity from small (1000–5000 head, *n* = 75, 12.6% of fed cattle) to medium (5001–10,000 head, *n* = 48, 24.3% of fed cattle) and large (>10,000 head, *n* = 48, 65.8% of fed cattle) [14]; data for Ontario feedlot bunk capacity were not publicly available. Cattle enter Canadian feedlots from just under 54,000 cow–calf farms [15], with some animals imported from the United States.

Most beef animals enter Canadian feedlots in the fall as recently weaned calves (450–600 lbs) that were born in winter or spring of that year, with some entering as higher-weight yearlings (approximately 900 lbs) [16,17]. Fall-placed calves are most often sourced from auction markets from multiple cow–calf farms and are considered at high risk for BRD [17]. In large, commercial feedlots in western Canada, calves and yearlings are typically managed in pens of 200–300 animals, with feeding time to slaughter at 1450–1700 lbs being 90–200 days, with a minority taking up to 300 days [16,17].

Feedlot veterinary practices are highly specialized entities that use digital technology, including proprietary integrated data management systems that are linked directly with their feedlot clients [18]. Feedlot veterinarians visit feedlots frequently (ranging from daily to monthly) to conduct postmortems, examine cattle for export, visit feedlot managers to review data, conduct outbreak investigations, train feedlot staff, conduct research trials, perform any required surgeries, and manage sick animals in the chronic pen.

Laboratory diagnostic tools are an important part of an AMS strategy in livestock production to provide information about the pathogens implicated in an infectious disease, along with associated AMR for bacterial infections. The ideal scenario would be to have rapid tests to inform antimicrobial decisions in individual animals at the time of administration. However, the current lag-time between sample collection, shipment, testing, and receipt of results means that in feedlot management of BRD, for example, samples are typically taken from live animals to provide group-level information as a part of research projects or surveillance programs [19,20,21,22]. Live animal samples from early in the feeding period and peak BRD incidence provide different and potentially more valuable information for managing disease than samples taken from mortalities treated with multiple rounds of antimicrobials. Recent work has mapped the potential for integrating laboratory test data from live animals as part of BRD management in western Canadian feedlots, with an intent to provide pen- and feedlot-specific information to refine antimicrobial treatment plans for BRD [16].

Using traditional culture and antimicrobial susceptibility methods for the testing of samples from live animals, veterinary laboratories have played an important role in the understanding of BRD pathogens and AMR in the beef industry [22,23,24]. Commonly reported laboratory methods also include molecular tests [25,26,27]. In the case of BRD, the availability of metagenomic tools capable of identifying multiple pathogens and genes associated with AMR from a single sample assay could provide a new strategy to support evidence-based AMU [28,29].

Optimal or prudent use of antimicrobials in animal health as well as sampling for laboratory testing depends on the vet–client–patient relationship [30]. In Canada, veterinarians assume a supervisory role over sample collection, processing, and shipping to diagnostic labs; typically, the tasks associated with BRD sample collection are performed by registered veterinary technologists employed by the veterinary clinics [22]. In some cases, sample collection is conducted as part of national surveillance programs. Surveillance data are then made available for use by other clients and stakeholders such as feedlot staff, managers, and owners as well as government entities [22].

Despite the long-standing work of the beef industry on AMS and AMR surveillance [8,20,22], a specific focus on the operational realities of existing feedlot practices regarding respiratory sample collection from live animals in support of BRD management is yet undescribed. This practical experience is a rich source of information about current perceptions of opportunities and barriers for respiratory sample collection and the potential value of laboratory information for BRD management in Canadian feedlots.

The overall goal of this research was to work with feedlot veterinarians (knowledge users) in Canada to understand the operational realities of the development and implementation of laboratory tools for BRD management and AMS in feedlots. The specific objectives of this study were to (1) identify factors that influence live animal respiratory sample collection for laboratory testing and (2) describe the potential for integration of laboratory testing of samples from live animals into an AMS strategy for BRD management in Canadian feedlots.

## 2. Materials and Methods

### 2.1. Study Design

Using focused ethnography [31], key informant interviews were conducted with the target population (Canadian feedlot veterinarians). Focused ethnography is a method of qualitative inquiry based on participant observation (i.e., where the researcher is immersed in the study setting or population) conducted within a particular context among a target group of people to inform decision-making regarding specific topics [31]. Collaborating Canadian veterinary practices that specialize in feedlot production medicine provided access to a network that includes the relatively small number of feedlot veterinarians and linked professionals responsible for the health and welfare of most of the fed cattle in Canada. The research team used their knowledge of the beef feedlot industry, their past and existing relationships with stakeholders, and internet searches to identify potential participants whose scope of practice was focused primarily on feedlot production medicine. Invitations were sent to 14 feedlot veterinarians within eight clinics from Alberta, Saskatchewan, and Ontario, which are the provinces accounting for over 89% of fed cattle in Canada [13]. These practices were identified based on the knowledge of the co-authors based on working relationships for research and surveillance. Each invited veterinarian was given an anonymous participant identification number prior to the interviews for tracking (e.g., PARTICIPANT001-014).

### 2.2. Ethical Approval and Consent to Participate

This study was approved by the University of Alberta Research Ethics Board (REB) (MS3_Pro00108325) on 19 July 2021, which was recognized at the University of Saskatchewan as a collaborator on the grant that funded this work. The right to informed consent was strictly observed during the study. A REB-approved letter detailing the consent process, study objectives, and estimated time required was sent to participants. Before each interview, informed consent was verbally confirmed with all participants, emphasizing their right to ask questions, refuse to answer, or withdraw from the study at any time during the interview. The complete application and informed consent letter are available upon request; the interview guide is included in Supplementary Materials.

### 2.3. Study Area

#### 2.3.1. Data Collection

A semi-structured interview guide (Supplementary Materials) was developed by one of the authors (O.O.A.) and revised to reflect feedback from the broad research team [32,33]. This feedback process included a specific review with a feedlot and beef research veterinarian and a veterinarian who specializes in AMR/AMU surveillance.

The interview guide was then tested with another feedlot research veterinarian and revised for use with study participants. The interview guide comprised five sections, covering questions related to the following topics: (1) current laboratory testing strategies to detect BRD pathogens and AMR, (2) type and strategy for collection of respiratory samples, (3) the role of veterinary and feedlot staff in collection of samples for laboratory testing for BRD and AMR, (4) the perceptions of participants about the role of laboratory testing to inform BRD treatment plans, and (5) the potential role of BRD laboratory testing in AMS to adhere to future AMS regulations.

The key informant audio interviews were conducted and recorded via Zoom^®^ [34]. A moderator (S.J.G.O.) led the discussion for all interviews, and two reporters (O.O.A. and another research assistant) took notes to supplement the audio recordings. The audio recordings were stored on a password-protected, encrypted project laptop, with routine back-up on the University of Alberta Google-enabled cloud-based storage system (Google-drive, Alphabet Inc., Mountain View, CA, USA). Interview recordings were sent securely to the University of Saskatchewan Canadian Hub for Applied and Social Research (CHASR) (Saskatoon, SK, Canada) for transcription. A sample of the transcription was validated and checked (O.O.A.) for completeness.

#### 2.3.2. Data Analysis

An inductive, non-linear, iterative, thematic analysis [35] was conducted (O.O.A.) in NVivo (Academic version 12, QSR International, Melbourne, Australia) [36]. An a priori codebook was developed (OOA) using the interview guide and then applied to each interview transcript. Descriptive codes were systematically and manually assigned to every line of the interview transcripts to label and identify ideas in the data that aligned with the research objectives. The connections and relationships observed between the different codes informed the generation of themes and sub-themes to describe the relayed experiences of participants relative to the study objectives. The preliminary findings of the study were subjected to peer debriefing by a member of the research team to obtain valuable feedback on the research process and outcomes and to strengthen credibility [31,37].

## 3. Results

Eight feedlot veterinarians from six practices in Alberta, Saskatchewan, and Ontario agreed to participate. Five of these practices represent all the specialty veterinary practices in Canada that focus exclusively on feedlot medicine and production (personal knowledge of the co-authors); one was a mixed-animal practice that also works with feedlots. The two practices that did not participate were mixed-animal practices that work with feedlots. The five specialized feedlot veterinary practices were responsible for managing the health and welfare of most of the fed cattle in Canada through a veterinary–client–patient relationship with the feedlot owners (personal knowledge of the co-authors; exact data not publicly available).

The duration of the interviews ranged from 42 to 81 min. Codes assigned to the data included (1) situations where veterinary clinic and feedlot staff collect live animal respiratory samples for laboratory submission and testing; (2) perceptions on levels of detail, accuracy, and timing of BRD laboratory results for consideration in BRD treatment plans; (3) the value of laboratory testing to inform AMS; and (4) AMS for market access.

**Figure 1:**
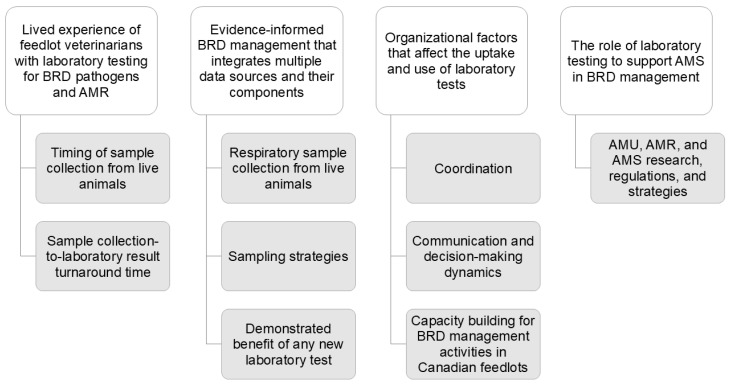
Themes (white blocks) and sub-themes (grey blocks) describing the perceptions of feedlot veterinarians about live animal respiratory sample collection and implementation of laboratory testing tools to support antimicrobial stewardship (AMS) in the management of bovine respiratory disease (BRD) and antimicrobial resistance (AMR) in Canadian feedlot cattle. AMU—antimicrobial use.

Four main themes and nine sub-themes were identified (Figure 1 and Table 1). These themes include (1) the lived experience of feedlot veterinarians with laboratory testing for BRD pathogens and AMR, (2) evidence-informed BRD management that integrates multiple data sources and their components, (3) organizational factors that affect the uptake and use of laboratory tests, and (4) the role of laboratory testing to support AMS in BRD management. These themes (and their sub-themes) were then used to describe the potential integration of laboratory testing into an AMS strategy for BRD management in Canadian feedlots.

### 3.1. Lived Experience of Feedlot Veterinarians with Laboratory Testing for BRD Pathogens and AMR

Participating veterinarians reported they and their staff had extensive experience in obtaining live animal respiratory samples from both BRD symptomatic and asymptomatic animals within feedlot settings. However, while they collected samples for laboratory testing to investigate BRD pathogens and AMR, the routine collection of respiratory samples from live animals was not part of their regular protocols for BRD management. This activity was predominantly undertaken in response to specific circumstances, such as the participation in research projects or surveillance programs sponsored by governmental bodies or industry stakeholders or to investigate disease outbreaks.

Participants described factors that influence the collection of respiratory samples and the possibility of using laboratory tools for BRD pathogen and AMR detection in Canadian feedlots to inform AMS. These factors were timing of sample collection and turnaround time from sample collection to laboratory report.

#### 3.1.1. Timing of Sample Collection from Live Animals

Participants discussed potential timepoints for live animal respiratory sample collection from feedlot cattle to be (1) at arrival processing, (2) 10–14 days on feed (DOF), (3) when sick cattle are first pulled and treated for BRD, (4) reimplantation (approximately 60–80 DOF), and (5) at terminal weight sorting (approximately 30 days prior to slaughter). They noted that timing of arrival processing can be unpredictable, creating challenges for coordination with the veterinarians and trained veterinary staff for sample collection. Arrival processing involves various activities, such as administration of antimicrobial metaphylaxis for BRD, retagging, implanting, vaccination, and parasite treatment, potentially increasing the difficulty of also integrating sample collection. Implantation is the placement of small, slow-release hormone boluses under the skin on the back of the ear [38]. These implants contain hormones that stimulate growth rate and feed efficiency, which are administered at arrival and again at 60–80 DOF. It is common practice for calves to receive vaccines at arrival, including for BRD pathogens, despite the uncertain benefit of BRD viral vaccines as identified in a systematic review and meta-analysis [39].

Participants explained how successful sample collection at arrival processing requires careful coordination and consideration of factors such as arrival day and time, processing timing, communication, and travel distance between the veterinary clinic and feedlots. Despite the shared logistic challenges, three participants suggested that sampling a subset of high-risk animals during arrival processing could support evidence-informed BRD treatment plans but not immediate decisions about antimicrobial metaphylaxis given the delay in obtaining laboratory results.

Participants highlighted that collecting respiratory samples at 10–14 DOF would be challenging, as animals are typically not handled at this time. They explained that this can be a time of peak BRD incidence and that handling animals for sampling could cause added stress and disease transmission opportunities. To justify sampling at 10–14 DOF, they noted there would need to be demonstrable benefit from the information gained. Six participants expressed interest in sampling at this time if improved health outcomes could be achieved. One suggestion was to consider sampling animals pulled for BRD treatment in the first 10–14 days rather than a random set of calves from a pen at that time. However, they acknowledged that this targeted approach could be biased towards animals sick with BRD and not representative of BRD pathogens and AMR circulating in the pen.

Routine handling for reimplantation (60–80 DOF) and terminal weight sort (approximately 30 days prior to slaughter) were identified as possible times for respiratory sample collection. However, as these two time points occur later in the feeding period, samples would not be representative of circulating pathogens and AMR during the period of high risk for BRD. In addition, participants noted that collecting respiratory samples from heavy cattle at terminal weight sorting increases the risk of animal and worker injury.

One participant explained the need to use supplemental restraint for heavy cattle to prevent injury to get good nasal or deep nasopharyngeal swab (DNPS) samples. This adds an additional cost and could negatively affect animal welfare. As a result, sampling at terminal weight sorting is largely not done.

#### 3.1.2. Sample Collection-to-Laboratory Result Turnaround Time

Turnaround time is the time from sample collection to the availability of laboratory results for veterinarians to make clinical decisions. Participants ideally desired a turnaround time that ranged from chute-side to 24 h to address the urgency of making antimicrobial administration decisions at the group level for BRD. Laboratory testing to inform drug decisions for individual animals in the chute would require test results in seconds, which participants acknowledged is not imminently available. Making pen-level decisions about BRD treatment plans for current animals would ideally require turnaround times of 24 h or less to be the most useful for that pen.

Participants explained that one factor causing delays in laboratory turnaround time is the time required to submit and ship samples from the feedlot or clinic to the laboratory. Measures like online laboratory submission and courier access simplify the submission process, but they do not mitigate long travel distances between the feedlots, clinics, and commercial laboratories, particularly in many areas of rural Canada. Weekend disruptions leading to sample transportation delays create further challenges.

In addition to transport time, the laboratory processing time required to complete and report the current standard bacterial culture and antimicrobial susceptibility testing was identified as an issue. The typical reported turnaround time for laboratory testing to detect BRD pathogens and test antimicrobial susceptibility from clinical samples exceeds four days, which participants did not find useful to inform BRD treatment plans. Participants emphasized the need for quick turnaround time for laboratory results to help inform management decisions and potentially add value to other data sources.

### 3.2. Evidence-Informed BRD Management That Integrates Multiple Data Sources and Their Components

Participants reported that they consider multiple data sources retrospectively to inform the BRD management activities and protocols for subsequent years. Some sources they consider include gross postmortem information, access to records for individual feedlots through their electronic records management system, discussions with the feedlot staff and owners, research results, Canadian Integrated Program for AMR Surveillance (CIPARS) results, and electronic access to laboratory results from clinical submissions. They obtain treatment, morbidity, mortality, and AMU/AMR data from these sources as well. The reported types of analysis obtained from these sources were quarterly and yearly group-level summaries for pens, feedlots, and arrival cohorts. The analysis of historic research and surveillance data were mentioned as potentially useful to inform BRD management and related plans for future production.

#### 3.2.1. Respiratory Sample Collection from Live Animals

All participants indicated that live animal respiratory sample collection and laboratory testing for detection of BRD pathogens and AMR is currently part of the research, surveillance, and outbreak investigation in Canadian feedlots. The sample types most commonly mentioned were DNPS and nasal swabs. Most commented on the potential for uncertainty in the consistency of results of upper versus lower respiratory samples. They also expressed the lack of practicality for collecting a “deeper” sample (e.g., bronchoalveolar lavage (BAL) or transtracheal wash (TTW)) as part of routine sampling in a commercial feedlot.

Some feedlot veterinarians collected respiratory samples from groups of calves during BRD outbreak investigations. They acknowledged that arrival metaphylaxis can alter the respiratory microbiome of feedlot calves, thus influencing the relative detection of bacterial BRD pathogens and the interpretation of antimicrobial susceptibility profiles.

#### 3.2.2. Sampling Strategies

Participants suggested sample sizes ranging from 5 to 10% of a group of calves, ranging from 5 to 30 animals in a group such as a pen, or the pooling of samples based on clinical assessment could be useful for understanding BRD at the group level. Most participants preferred population- or group-level laboratory data (e.g., at the level of the arrival cohort, pen, or feedlot) compared to individual calf data. Some reported situations where individual-level data (e.g., single animals with chronic BRD) might provide relevant information. Participants suggested that individual data could give an idea of pathogens responsible for an outbreak but are not necessarily representative of BRD at the pen or feedlot level. Also, participants mentioned the time to results and practicality as reasons to analyze and use pen- and feedlot-level data for AMS decisions in terms of feedlot-level BRD treatment plans. One participant specifically mentioned that sampling a small group of animals during the first 7–14 DOF (the high-risk period for BRD) might give results representative of the group while minimizing the stress from having to sample every animal in a pen or group:

“…*if we’re trying to manage individual animals, then we’re going to need individual results. If we’re going to try and manage pens, then we can use subsampling of some sort to predict or to be the proxy for what we need to do with the pen*.”

—PARTICIPANT001

#### 3.2.3. Demonstrated Benefit of Any New Laboratory Test

Participants consistently mentioned the need for useful laboratory tools to inform evidence-based treatment decisions, provide insights to curb outbreaks, and achieve research and surveillance objectives. Participants said any new laboratory test would need to show benefits above the current visual and clinical assessment, postmortems, or other existing laboratory methods (e.g., traditional culture and susceptibility testing) to justify the extra time, labor, and cost to the feedlot, for example, tests that would predict BRD outcomes. They acknowledged the practical considerations associated with the implementation of new laboratory tools. They also anticipated an increase in costs for the industry with new tests, which could ultimately be transferred to consumers:

“*The other possibility is, if we have a test… that’s predictive of outcome under our treatment regime…, then certainly we could justify handling those animals even at a slower speed, if we were going to get a better outcome…*”

—PARTICIPANT002

Participants noted that the current data from laboratory testing, such as that received for feedlots participating in the national surveillance program, are useful for retrospective assessment of BRD but not for real-time BRD treatment decisions. They emphasized the need to have laboratory information for the individual feedlots within their clinical practice to be useful for constructive communication with their clients:

“*We receive two reports. We receive a national report that is de-identified, that we use to have a look at the trends nationally in the [BRD] organisms that we’re [interested in]. And then, we have an individual producer report that is sent to us from CIPARS, and that tells us about what organisms have been found, and the antimicrobial resistance in those organisms. And we then pass that on and discuss that with our producers.*”

—PARTICIPANT003

Questions about the accuracy of laboratory data evoked responses about how these results would reliably inform relevant BRD disease prevention, investigation, or treatment decisions. The responses spoke to clinical outcomes at the pen- and feedlot-level as opposed to specific thoughts on the accuracy of laboratory tests at the level of the individual animal. They explained that laboratory tests must support the responsibility of the feedlot veterinarian to feedlot owners/management in terms of reducing BRD morbidity/mortality, improving animal welfare, and reducing the cost of production:

“*That’s our business model. We’re not going to recommend anything to our clients, particularly on a large scale, unless we have data that show that it is going to help them to identify, treat, and ultimately save more animals.*”

—PARTICIPANT003

### 3.3. Organizational Factors That Affect the Uptake and Use of Laboratory Tests

#### 3.3.1. Coordination

Participants expressed concern about the potential for feedlot staff complaints linked to the added work of respiratory sample collection from live animals at busy processing times contributing to burn-out and labour shortages. Staff frustration can also arise when animal processing times are increased due to sample collection. Extended processing times directly increase costs in staff wages and could lead to additional production losses from increasing animal handling stress:

“*From a coordination perspective, I guess you’d say probably that’s a [time of day] problem. When are the cattle coming into the feedlot? Is it during the day? Is it during work hours? That type of thing. And do they want to slow down a bit to allow you to take those samples? Because it’s going to definitely take a little bit more time to do that than it would take to just do a normal processing procedure. It is pretty quick. If you were to do every single animal, you couldn’t keep up.*”

—PARTICIPANT011

#### 3.3.2. Communication and Decision-Making Dynamics

Participants described the multi-stakeholder interaction between feedlot veterinarians and the feedlot owners, managers, and staff. These interactions result in decisions that are not only driven by science, but are also aligned with the broader objectives and operational perspectives of the feedlot.

The process of engaging in research or surveillance involves more than the recommendation by feedlot veterinarians; it necessitates collaborative decision making with their clients. The dynamics of this partnership were described by study participants who underscored the importance of effective communication with the feedlot management team. One participant used the term “sell” (Table 1) to convey the depth of communication necessary for surveillance and research activities in client-owned feedlots. Such communication entails conveying the technical aspects and potential benefits and implications of the proposed activities to solicit buy-in by clients.

#### 3.3.3. Capacity Building for BRD Management Activities in Canadian Feedlots

Participants identified the existing capacity for respiratory sample collection to support laboratory testing for research, surveillance, and clinical investigation. Collaborators who could be involved with sample collection included veterinarians, registered veterinary technologists/technicians, non-registered veterinary technical staff, foreign-trained veterinarians (conditionally licensed in Canada), research project managers, cattle managers, feedlot processing crews, pen checkers, and other feedlot staff. Factors contributing to the selection of collaborators for respiratory sample collection were skills, training on standardized operating procedures, and motivation. Registered veterinary technicians were most commonly delegated to collect samples with veterinarians either supervising directly on-site or through remote communication:

“*We’ve all been trained and a lot of that was through the protocols through the research projects we’ve been involved with. And then we do—we train the staff*.”

—PARTICIPANT004

Participants reported that feedlot staff have shown enthusiasm for activities outside of routine processing, such as respiratory sample collection. Participants train feedlot staff to collect respiratory samples to further support access to feedlot resources, cost reduction, and better coordination of research activities. Other activities delegated to feedlot staff are data collection, accurate sample labelling, sample handling, and packaging for shipping:

*[Feedlot staff are motivated to work outside the routine to] “expand and improve their skillsets of things that they can do, [because] some of the things that they do are repetitive and maybe not as mentally challenging as they could be, just because it’s the nature of the work.*”

—PARTICIPANT001

### 3.4. The Role of Laboratory Testing to Support AMS in BRD Management

#### 3.4.1. AMU, AMR, and AMS Research, Regulations, and Strategies

The consensus among participants was that the integration of BRD laboratory data from proven testing strategies could improve AMS. This underscores the collective recognition that to preserve the effectiveness of antimicrobial agents and to meet evolving market and regulatory expectations, there needs to be a viable pathway to evaluate existing AMU protocols relative to laboratory data, animal health outcomes, and future AMS regulations.

Participants also identified that having a sampling and laboratory testing strategy that aligns with feedlot management is important to inform which antimicrobials are used as part of BRD management. They are interested in laboratory testing strategies that could inform targeted treatment to specific animals and groups. This could lead to cost savings for producers and continue to maintain the trust of consumers.

A key challenge that was identified is the need to convincingly demonstrate to feedlot owners and operators the economic and animal health outcome advantages of integrating new laboratory tools within the existing management frameworks. However, the participants anticipated that future regulations might mandate the use of laboratory evidence to substantiate the diagnosis, necessity, and selection of antimicrobials for treatment:

“*Laboratory testing seems to be the ticket to get us there [export markets], and so we’re very interested in that. Chute-side laboratory [tests] with targeted treatment, I think, [would] do an excellent job of balancing animal health and welfare concerns, and antimicrobial use and resistant concerns, in my opinion.*”

—PARTICIPANT002

## 4. Discussion

This study documented the perceptions of Canadian feedlot veterinarians regarding live animal respiratory sample collection and using laboratory testing tools to inform BRD management in feedlots. Feedlot veterinarians were willing to incorporate laboratory testing of live animal samples to provide pen-level data and potentially at new times (e.g., 10–14 DOF) if these strategies translate into improvements in BRD outcomes. They also appreciated the potential importance of laboratory testing strategies to support AMS, particularly with the possibility of future changes to AMS regulations that could require laboratory testing. These findings align with our recently published strategy that considers pen-level sampling and testing of live animals at arrival and 10–14 DOF (peak BRD incidence) to provide BRD and AMR data to inform pen- and feedlot-level BRD treatment plans in western Canadian commercial feedlots [16].

This study confirmed the wealth of experience that exists within Canadian feedlot production for BRD pathogen and AMR surveillance and research [22,40]. The feedlot veterinary participants highlighted the collaborative work with their staff and feedlot personnel to coordinate live animal sample collection for such efforts. However, these efforts are limited to retrospective assessment of data from surveillance, research, and outbreak investigations [41]. Laboratory testing of samples from live animals is not routinely used as part of antimicrobial metaphylaxis and treatment decisions for BRD management in Canadian feedlots. As a result, feedlot veterinarians appear uncertain whether feedlot staff, management, and owners will appreciate the benefits of laboratory testing to inform AMS and BRD management. Due to this uncertainty, feedlot veterinarians feel they have to “sell” laboratory testing to their clients.

Feedlot veterinarians identified that more timely laboratory information at the group level could help inform AMU as part of BRD management, in particular for identification of bacterial versus viral agents [42]. Advances in laboratory testing, such as third-generation metagenomic sequencing, show promise in achieving these goals with more comprehensive BRD organism and AMR gene detection capabilities [28,29].

This study revealed the interconnections between the scientific and ethical lenses through which Canadian feedlot veterinarians view the possible integration of laboratory testing for BRD. Ethically, the study findings reinforced the professional responsibility of veterinarians to safeguard animal well-being and minimize unnecessary suffering [43]. Scientifically, veterinarians seek evidence-informed interventions and strategies to mitigate BRD risks and enhance feedlot management and production.

Feedlot veterinarians explained that they prioritize both animal and veterinary clinic and feedlot staff welfare, which is supported by previous work [44]. Animal welfare aims to reduce losses due to disease, outbreaks, and injury [45,46,47,48]. Staff welfare includes initiatives such as improving coordination, communication, and capacity to optimize operational efficiency and promote work-life balance. This holistic approach not only ensures the well-being of animals but also enhances the overall health and morale of the workforce, ultimately contributing to the resilience and success of the feedlot industry.

Organizational factors such as capacity, coordination, and communication are crucial when considering how a laboratory test could effectively integrate into Canadian feedlots as a support for management of BRD and AMS. Coordinating respiratory sample collection at specific time points could pose challenges for veterinarians who might not always be aware of when cattle are arriving at feedlots. Additionally, feedlot veterinarians often manage multiple sites that could be large distances apart. Coordinating all respiratory sample collection events would be challenging and potentially create conflict with their clients (the feedlot owners, managers, and staff). These considerations underscore the importance of effective coordination, communication, and capacity building within the various staff roles in a feedlot veterinary practice and within a feedlot.

The feedlot veterinarians in this study identified the importance of communicating to feedlot owners, managers, and staff about the objectives of sampling and laboratory testing to support AMS strategies. Prioritizing efficiency and animal welfare in the process (e.g., the animal handling system and time for sample collection) with emphasis on open lines of communication and collaboration among the feedlot and veterinary staff would support this strategy and promote uptake.

Challenges of sample transportation to the laboratory, such as shipping delays and labeling/packaging errors, impede timeliness of results. The relative remoteness of the feedlots to clinics and urban centres for shipping continues to be a challenge. Finding solutions to the inefficiencies of sample transport might improve the timeliness of results and promote uptake of strategic live animal testing for AMR in the feedlot sector.

Existing live animal respiratory sample collection for surveillance is typically incorporated into common animal handling time-points, namely processing at arrival and re-implantation (60–80 DOF). Sampling at re-implantation occurs after the time of peak BRD incidence and AMR transmission, making laboratory data from this time point less informative for BRD management and AMS [23,24,49]. Data obtained from sampling at 10–14 DOF may provide more actionable insights for BRD and AMS. However, the potential animal welfare, operational, and financial burdens require justification based on evidence of benefit given that it is inconvenient to handle animals at this time.

Identifying other management and BRD-related handling opportunities to coincide with earlier sample collection from live animals could offer potential advantages and reduce associated operational limitations. For example, either delayed respiratory vaccination or revaccination offers an opportunity for respiratory sample collection between 10–30 DOF rather than at arrival processing. A study comparing auction-market calves vaccinated at arrival processing versus at 30 DOF found that delayed respiratory vaccination reduced the risk of requiring multiple treatments for BRD [50]. Another study showed improved performance and reduced BRD-related issues when high-risk calves were vaccinated at 14 DOF [51]. However, a review suggested no significant differences in health outcomes with delayed respiratory vaccination overall, but noted that more research is needed, particularly outside of the U.S., to assess its effectiveness in different contexts like Canadian feedlots [52].

Feedlot veterinarians in this study preferred upper respiratory tract samples (i.e., nasal swabs and DNPS) over lower respiratory tract samples (i.e., TTW or BAL) for investigating BRD and AMR. Lower respiratory tract samples are impractical in commercial feedlots due to the time, sedation, and local analgesia required [53]. Current CIPARS surveillance for BRD in Canadian feedlots relies on DNPS [20]. Participants expressed concerns about potential discrepancies between upper and lower respiratory tract sample results for detecting BRD pathogens and AMR. However, the sampling and testing of a subset of most healthy cattle does not require agreement between DNPS and LRT samples because healthy cattle are not expected to have pathogens in their lungs [16]. Upper respiratory swabs from a representative sample of calves provide insights into the BRD pathogens and AMR circulating in a pen.

Feedlot veterinarians preferred sampling a representative subset of animals from a typical group, such as an arrival lot or pen rather than individual animals when sick. They identified that results from individual animal sampling are not quick enough to have any bearing on treatment decisions for those animals. This aligns with the broader understanding of BRD risk assessment for calves on arrival, in that current algorithms that assign groups of calves to high or low risk for BRD more accurately predict BRD risk at the group level than the individual [8,54,55]. The current state of laboratory tests that investigate BRD pathogens and AMR are not such that they can inform individual-level animal treatment decisions; however, there is promise in group-level testing to inform BRD treatment protocols [16,24,56]. The use of representative group-level samples provides some needed simplicity in that not all animals must be handled for sampling, and useful laboratory data can be applied to larger groups of calves early in the feeding period.

Ultimately, feedlot veterinarians want to know that a sampling and laboratory testing strategy will result in demonstrable improvements in BRD management and health outcomes. When asked about test accuracy, participants did not highlight the epidemiological importance of sensitivity/specificity and positive/negative predictive values [57]. Rather, participants tended to identify their role and responsibility to provide effective care to their patients and effective BRD management strategies to feedlot staff and ownership. This speaks to the fact that veterinarians integrate these epidemiologic principles into their broader decision making alongside these overall goals of production and animal health and welfare.

Feedlot veterinarians ideally need to obtain more timely results for BRD management than what retrospective data from traditional culture and antimicrobial susceptibility testing for research and surveillance currently offer. However, chute-side tests for AMR are not imminently available to inform real-time BRD treatment at the level of the individual or groups of animals [58].

This study provided insights into how feedlot veterinarians view laboratory testing relative to possible future regulatory or industry requirements for laboratory evidence to justify AMU for management of BRD. The WHO [5] and the European Union [59] at the international level as well as McDonald’s [60], as one of the largest customers for the Canadian beef industry, are moving towards requirements for laboratory evidence to support AMU decisions for food animals. In addition, the Canadian province of Québec enacted a regulation in 2019 requiring laboratory evidence to justify the use of category I antimicrobials [61] in food producing animals for treatment and prohibits their use for disease prevention [62].

Feedlot veterinarians recognize the importance of AMS and are interested in the possibility of new laboratory strategies to support stewardship as long as they align with the objectives and priorities of the feedlot operations of their clients. The commitment of Canadian veterinarians to AMS through research and surveillance [20,22] underscores the opportunity for integrated approaches that combine improved animal management practices with laboratory testing [16]. These strategies could contribute to supporting animal health and welfare, economic viability for producers, and the mitigation of AMR risks.

Using focused ethnography as the research method in this study has limitations, such as the scope being restricted to feedlot veterinarians. The feedlot staff, veterinary staff, and laboratory contexts and inter-relationships represented in this study were not fully captured due to this specific focus. Future studies could include other members of the feedlot veterinary practice team and feedlots themselves. Yet, the strength of using focused ethnography was in elucidating the perspectives of feedlot veterinarians as people positioned to influence change within the industry. Veterinarians are responsible for maintaining the veterinary–client–patient relationship and for prescribing antimicrobials and providing and updating BRD management protocols to their feedlot clients. Further research could explore cost implications as part of the value proposition for laboratory testing to demonstrate the potential economic costs, benefits, and viability of implementing sustainable antimicrobial stewardship strategies.

## 5. Conclusions

This study described the perceptions of Canadian feedlot veterinarians on the factors that influence laboratory testing of respiratory samples and subsequent integration of BRD pathogen and AMR data to support AMS in Canadian feedlots. The findings highlight that while sample collection and laboratory testing are commonly used in commercial feedlots for BRD research and surveillance, they are not routinely applied in everyday BRD management. Veterinarians identified key challenges with laboratory testing, including the need for clear benefits, practical implementation, and effective communication with feedlot managers. Laboratory testing must provide valuable, actionable insights to encourage its adoption in Canadian feedlot operations. If successful, such testing could support AMS and help to meet future regulations or market demands. This research supports responsible antimicrobial use in food animal production, which can help reduce the risk of AMR and improve animal health and welfare. By gaining insights into the lived experience of Canadian feedlot veterinarians, this study contributes to the broader conversation about utilization and optimization of laboratory testing as an AMS strategy in food animal production.

## Figures and Tables

**Table 1:** Themes, sub-themes, and selected quotes describing the perceptions of feedlot veterinarians about live animal respiratory sample collection and implementation of laboratory testing tools to support antimicrobial stewardship (AMS) in the management of bovine respiratory disease (BRD) and antimicrobial resistance (AMR) in Canadian feedlot cattle. AMU—antimicrobial use.

Theme	Sub-Themes	Example Quotes from Respondents
3.1. Lived experience of feedlot veterinarians with laboratory testing for BRD pathogens and AMR	3.1.1. Timing of sample collection from live animals	*“We’ve done arrival sampling, and that happens basically at induction to the feedlot. When the group of animals is being put through the handling facility and given their vaccines or other medications we would collect a nasal swab at that time. The other time that we’ve done it has been at that first [BRD treatment] pull, just to see what organisms or what their [antimicrobial] sensitivity would look like in those animals.”* —PARTICIPANT007
	3.1.2. Sample collection-to-laboratory result turnaround time	*“I want the results right now…. That’s the only way it’s going to be useful, because I have to treat that animal in the chute right now. I’m not running them back through…” —*PARTICIPANT008*“To have a specific test that could be used to identify BRD, particularly chute side, you get an answer right then, it doesn’t even need to go to a lab. I think that is ultimately what would make the biggest difference.”* —PARTICIPANT003
3.2. Evidence-informed BRD management that integrates multiple data sources and their components	3.2.1. Respiratory sample collection from live animals	*“I’m going to say, a deep nasal pharyngeal swab. They’re not transtracheal washes or BALs [bronchoalveolar lavage]. We haven’t gone to those lengths, at this point. I know there’s probably pros and cons to those, but practically speaking, the deep nasal swab has worked well for us.”* —PARTICIPANT007
	3.2.2. Sampling strategies	*“If you’re looking at sampling, part of that’s going to be part of the size of the group that’s coming in, so there’s probably a few factors. But statistically, I usually go for 10 or 12 [animals in a group] if I can, as my minimum.”* —PARTICIPANT007.
	3.2.3. Demonstrated benefit of any new laboratory test	*“I think it ties back to the accuracy. The lab gives us information that information seems to be helpful in either predicting disease or helping to reduce your morbidity and your mortality rates… . You’re going to use your experience with that…”* —PARTICIPANT003
3.3. Organizational factors that affect the uptake and use of laboratory tests	3.3.1 Coordination	*“If management, whether that’s the immediate supervisor, or the person above that, or the owner-manager, if they’re not onside with the sample collections systems or doing it [sample collection], it actually simply won’t work. Because there’s too many conflicting messages that are subsequently filtered down to the frontline folks.”* —PARTICIPANT001
	3.3.2. Communication and decision-making dynamics	“*I had to sell research to my clients [feedlot owners].”—*PARTICIPANT007*“Some of it is strictly related to research. We do a contract research project, there may be a company that just wants to know what organisms they are seeing before or after treatment.”* —PARTICIPANT002
	3.3.3. Capacity building for BRD management activities in Canadian feedlots	*“People that are in [feedlot] management or have an interest in what you’re doing, I think, is a big help. And then, [they have] to be able to understand or comprehend [the reason for sampling and testing], but if you’ve got all that together, it’s worked well for us.”* —PARTICIPANT007
3.4. The role of laboratory testing to support AMS in BRD management	3.4.1. AMU, AMR, and AMS research, regulations, and strategies	*“We use it to make decisions on our antimicrobial use protocols. And so, it’s not as much for how we fit into the prudent use guidelines with the different classes of antibiotics, because that’s already set. But it really is [about] what are our choices of antibiotics are going to be within those different classes. And when and if we’re going to change our antimicrobials.”* —PARTICIPANT004

## Data Availability

Dataset available on request from the authors.

## Supplementary Materials

The following supporting information can be downloaded at https://www.mdpi.com/article/10.3390/vetsci12050409/s1.

## Abbreviations

The following abbreviations are used in this manuscript:

AMS	Antimicrobial stewardship
BRD	Bovine respiratory disease
AMR	Antimicrobial resistance
AMU	Antimicrobial use
WHO	World Health Organization
REB	Research Ethics Board
CHASR	Canadian Hub for Applied and Social Research
DOF	Days on feed
CIPARS	Canadian Integrated Program for AMR Surveillance
DNPS	Deep nasopharyngeal swabs
BAL	Bronchoalveolar lavage
TTW	Transtracheal wash
