# Supplementary material for: Laboratory Testing to Inform Antimicrobial Use for Bovine Respiratory Disease: Perceptions of Canadian Feedlot Veterinarians
Source: Vet Sci. 2025 Apr 27;12(5):409. doi: 10.3390/vetsci12050409 (PMC12115976; doi:10.3390/vetsci12050409)
Supplement: Supplementary file 1 [file vetsci-12-00409-s001.zip › vetsci-3549877-supplementary.pdf]

List of Interview Questions

A. Respiratory sample collection:

The first set of questions focuses on situations where you or your clinic staff have collected respiratory samples from live animals, specifically deep guarded nasopharyngeal swabs or superficial nasal swabs.

1. Do you currently send nasal swabs to a diagnostic laboratory for antimicrobial susceptibility testing for BRD pathogens? If so, how frequently? If not, please tell us why not.
2. Have you or your staff collected respiratory samples from live animals for any of the following situations:
    - Clinical laboratory diagnostic testing on sick individual live animals?
    - Clinical laboratory testing on individual live animals to provide pen- or group-level information? (e.g., pen outbreak)
    - Research projects?
    - Surveillance programs (e.g., CIPARS)?
3. Are there any other scenarios, and if so, please describe when you would collect respiratory samples?
4. (If yes to any of the above, then ask these questions):
    a) When are these samples collected? (on arrival, when pulled for suspect BRD, other?)
    b) What types of samples are collected? (type of swab, media, deep nasopharyngeal swabs, superficial swabs, or not)
    c) Where do you submit samples? (which lab)
    d) How are these samples submitted for analysis? (Courier?)
    e) What are the protocols for shipping samples to the lab?
    f) Who in your practice collects the samples?
    g) Please describe how you use the information obtained from the lab about these samples.
5. (*If there are clinic staff collecting these samples*) Are the people in your clinic collecting samples trained to do so? If so, how are they trained? How are they supervised?

B. Challenges with collecting respiratory samples:

6. Have you experienced any challenges with coordinating collection of samples at arrival? (If answer is NO skip to next section)
    - If so please describe these challenges and how you have resolved them.
7. Is there a scenario where animals can be sampled shortly after a short period of time on feed? If yes, please describe it and how can this be done to minimize challenges?
    - If not, please describe the challenges of this approach or a more feasible alternative.
8. Are there other challenges when collecting samples for BRD from animals later in the feeding period that you have not described above? If so, please describe them.

C. Situations where feedlot staff are collecting respiratory samples for laboratory submission:

9. Do you use feedlot staff to collect respiratory samples from live animals for diagnostic lab submissions? (If not skip to the next section.) If so please describe these situations. If not, why not?
10. What situations worked well for feedlot staff to collect samples? Please describe these situations.
11. Have you had challenges with feedlot staff collecting respiratory samples? If so please describe them.

D. Laboratory Diagnostics and treatment plans for BRD:

12. Does laboratory diagnostic information currently have a role to inform the establishment of feedlot or pen specific treatment and disease management protocols for BRD? If so, please describe.
13. In your experience, are there any barriers to the routine application of laboratory diagnostic testing for informing management of BRD? If so, what are they?
14. What is the longest that you can wait to get lab information back to inform BRD treatment decisions for a pen that is sick right now?
15. What is the longest that you can wait to get lab information back to inform BRD treatment decisions for future sick animals or pens?
16. Do you compile laboratory information from a feeding period to inform management of BRD for future feeding periods? If so, please describe how you use this information.
17. What degree of detail do you need from laboratory diagnostic information to manage BRD?
    - individual animal?
    - pen/group level results?
    - if groups, how many animals in a group need to be sampled?
    - do you need information from a single test in time, or repeated tests over time on specific animals or pens of animals?
18. With metagenomic testing, we can potentially provide additional details about BRD and AMR within 24 hours. What additional details do you want to inform BRD management?
    Prompts:
19. Do you consider the accuracy of the laboratory information you receive of respiratory samples?
20. What is the optimal level of accuracy you would like to have from laboratory diagnostic information?
21. Is the source of test information important for you in order to trust the laboratory diagnostic data? If so, what sources do you feel are appropriate for the western Canadian context?
22. Is the source of accuracy about the test information important for you in order to trust laboratory diagnostic data? If so, what sources do you feel are appropriate for the western Canadian context?
23. Finally, regarding the quality of information, please describe any other factors we have not talked about that influence your decision to use or not to use laboratory diagnostic testing to manage BRD.

E. Antimicrobial stewardship for market access:

24. Could BRD laboratory diagnostic information enhance antimicrobial stewardship? If so, please describe how? If not, why not?
25. If WHO or EU recommendations to use laboratory diagnostic testing to justify antimicrobial use in food animals became a requirement, what, if any, potential benefits do you see for your practice, your clients, and/or the broader industry?
26. Alternatively in this scenario, what are the costs and challenges to your practice, clients, and/or the broader industry of having to obtain this information to access antimicrobials?

F. Insights:

27. Based on your experience, is there anything else you would like to add about sampling and/or laboratory diagnostic testing for BRD?
